# Monoclonal Antibody-Based Sandwich ELISA for the Detection of Staphylococcal Enterotoxin A

**DOI:** 10.3390/ijerph10041598

**Published:** 2013-04-19

**Authors:** Hua Kuang, Wenbing Wang, Liguang Xu, Wei Ma, Liqiang Liu, Libing Wang, Chuanlai Xu

**Affiliations:** 1 State Key Laboratory of Food Science & Technology, School of Food Science & Technology, Jiangnan University, Wuxi 214122, China; E-Mails: wenbin66@yeah.net (W.W.); xuliguang2006@yahoo.com.cn (L.X.); mawei209@126.com (W.M.); raxray@gmail.com (L.L.); xcl@jiangnan.edu.cn (C.X.); 2 Research Centre of Hunan Entry-Exit Inspection and Quarantine Bureau, Changsha 410001, China; E-Mail: wanglb1@126.com

**Keywords:** staphylococcal enterotoxin A (SEA), monoclonal antibody, sandwich ELISA, detection

## Abstract

A sensitive and specific monoclonal antibody-based sandwich enzyme-linked immunosorbent assay (ELISA) was established and validated for the detection of staphylococcal enterotoxin A (SEA). After routine fusion and selection, 10 monoclonal antibodies showed high affinity for SEA. An optimal pair for sandwich ELISA was selected by pairwise interaction analysis. After optimization, the limit of detection (LOD) and linear dynamic range of the method were established, and were found to be 0.0282 ng/mL and 0.06–2 ng/mL, respectively. The recovery in pure milk ranged from 82.67% to 111.95% and the intra- and inter-assay coefficients of variation ranged from 3.16% to 6.05% and from 5.16% to 10.79%, respectively. Cross-reactivity with staphylococcal enterotoxin B (SEB), staphylococcal enterotoxin C (SEC), staphylococcal enterotoxin D (SED), and staphylococcal enterotoxin E (SEE) in this method were insignificant. These results indicate that the sandwich ELISA method developed in our study is effective for routine identification of SEA in food samples.

## 1. Introduction

*Staphylococcus aureus*, a well-known food-borne pathogen, causes staphylococcal food poisoning (SFP) by producing enterotoxins in food such as meats and dairy products [1,2]. Staphylococcal enterotoxins (SEs) are single-chain proteins with molecular weights ranging from 27 to 34 kDa [3]. SEs are heat-stable, resistant to gut proteases, and active over a wide pH range [4]. Symptoms usually associated with SFP include nausea, vomiting, abdominal cramps, and diarrhea [5]. The actual toxic dose of SEs is still not very clear, although it was reported that 200 ng or less of SEA can produce illness in sensitive individuals [6]. Moreover, research on some SFP outbreaks found that SEA was detected at a concentration around 0.5 ng/mL [6,7]. Therefore, highly sensitive and reliable methods for analyzing SEs in food samples are critical for monitoring food products and traceback investigations in SFP outbreaks.

Numerous papers on methods for detecting SEs in food samples have been published [8,9,10]. Although to date there are more than 20 SEs and new SEs are being identified [11,12], the most common SE encountered in food poisoning outbreaks is staphylococcal enterotoxin A (SEA) [13,14,15].

During the last 20 years, several methods have been developed for the identification of SEA. These methods include polymerase chain reaction (PCR), mass spectrometry (MS), biosensor-based techniques, reversed passive latex agglutination (RPLA), immunoblotting, and ELISA [8,16,17,18,19,20].

PCR is a highly sensitive and rapid method. As little as only one to 13 copies of the *S. aureus* genome that contain a copy of the *entA* gene encoding the SEA protein can be detected by real-time PCR [17], but PCR cannot directly detect SEA and assess its levels in food. Nevertheless, it remains the preferred technique for confirming the presence of SEA-producing *S. aureus* strains in food samples.

MS-based methods have the advantage of high accuracy and repeatability in detecting proteins. Liquid chromatography-mass spectrometry and matrix-assisted laser desorption ionization time-of-flight mass spectrometry have been successfully used to detect SEA in food samples [8,21,22]. Although they are reliable and accurate, the LOD of these methods rarely reach <1 ng/mL. Moreover, the complexity of typical food matrixes renders sample preparation for these methods time-consuming and labor-intensive.

Analytical methods using biosensors for SE detection have been widely investigated because of their potential for realizing point-of-care applications in food poisoning outbreaks. Pimenta-Martins *et al.* [23] developed a method to detect SEA in cheese using an amperometric immunosensor; its LOD was found to be 33.9 ng/mL. A quartz-crystal microbalance immunosensor has been fabricated to assess SEA in a model medium buffer; the working range and LOD for the analysis based on this sensor were found to be 50–1,000 ng/mL and 20 ng/mL, respectively [18,19]. A surface plasmon resonance biosensor has been reported to detect 1–40 ng/mL (ppb) SEA in raw whole eggs [6]. Jantra *et al.* described a method for detecting SEA in various food samples based on a flow-injection capacitive immunosensor, and determined its LOD to be 1 fg/mL [3]. The immunosensor they used is highly sensitive and the matrix effects could be easily eliminated by dilution. However, the aforementioned sensor-based methods remain feasible only in a laboratory context and are not sufficiently mature to be adopted commercially for routine analysis.

Di Pinto *et al.* compared RPLA and immunoblotting in terms of their capabilities in confirming SEs in culture filtrates of a *S. aureus* strain [16]. RPLA is a relatively simple method for routine monitoring compared with immunoblotting, which is the standard method. However, these methods may be insufficient in detecting SEA in food samples.

ELISA has been considered as the most practical and powerful method for the analysis of SEA in foods because it affords sensitive and reliable results without relying on sophisticated equipment [24,25,26]. ELISA methods based on monoclonal antibodies (mAbs) provide results that are more reproducible than those obtained by methods utilizing polyclonal antibodies because mAbs are highly identical and specific. Moreover, mAbs are more suitable for commercial applications because they are a renewable source. Recently, a mouse polyclonal antibody-based sandwich ELISA has been reported to detect SEA in milk and cheese at concentrations as low as 0.064 ng/mL [13]. Although this method is very sensitive and specific, the polyclonal antibody used in this method may limit its industrial applications, so a highly sensitive and specific mAb-based sandwich ELISA for SEA remains to be realized, therefore, in the present study, we have developed a highly sensitive and specific mAb-based sandwich ELISA to detect SEA in milk samples.

## 2. Experimental Section

### 2.1. Chemicals and Materials

SEA, SEB, SEC, SED and SEE were purchased from the Academy of Military Medical Sciences (Beijing, China). Complete and incomplete Freund’s adjuvant and enzyme immunoassay-grade horseradish peroxidase (HRP)-labeled goat anti-mouse immunoglobulin were obtained from Sigma (St. Louis, MO, USA). Gelatin was obtained from Beijing Biodee Biotechnology Co., Ltd. (Beijing, China). 3,3',5,5'-Tetramethylbenzidine (TMB) and horseradish peroxidase (HRP) were purchased from Aladdin Chemistry Co., Ltd. (Shanghai, China). Pure milk was purchased from a local supermarket. All other reagents and chemicals were obtained from the National Pharmaceutical Group Chemical Reagent Co., Ltd. (Shanghai, China).

### 2.2. Solutions

The following solutions were used in this study: coating buffer (0.01 M sodium carbonate buffer, pH 9.6), blocking buffer (0.2% (w/v) gelatin in coating buffer), 0.01 M phosphate-buffered saline (PBS, pH 7.4), washing buffer (PBS containing 0.05% (v/v) Tween 20), antibody dilution buffer (PBS containing 0.1% (w/v) gelatin and 0.05% (v/v) Tween 20), stop buffer (2 M sulfuric acid), and substrate solution. The substrate solution was prepared by mixing a 2 mL solution of 0.06% (w/v) TMB in glycol with 10 mL of 0.1 M citrate phosphate buffer (pH 5.0) containing 1.8 μL of 30% hydrogen peroxide.

### 2.3. Antibodies and Conjugated Antibodies

The mAbs were prepared through a modification of a literature method [10]. First, the mice were immunized by a normal subcutaneous procedure using a series of three doses. The sequence of doses was 10, 10, and 5 μg recombinant SEA. After the three immunizations, the immune responses of the mice were measured by indirect ELISA. The mouse with the highest titer was sacrificed, and its spleen was fused with Sp2/0 murine myeloma cells. The target cells were selected by indirect ELISA and obtained by limiting dilution. The mAbs were purified by the caprylic acid-ammonium sulfate precipitation method and then conjugated to HRP, as described previously [9]. Briefly, the hydroxyl groups on HRP were oxidized to aldehyde groups by 0.06 M NaIO_4_ at 4 °C. Excess NaIO_4 _in the mixture was then eliminated by addition of 0.16 M glycol at room temperature. After this, purified antibody was added and the pH was adjusted to 9.0 with 0.05 M carbonate buffer. Amino-groups on the antibody reacted with the aldehyde groups on HRP and produced the corresponding Schiff bases. After incubating 20 h at 4 °C, stable conjugated antibodies were obtained by adding 5 mg/mL NaBH_4_ solution to the mixture. In the end, antibodies that conjugated with HRP were precipitated by addition of an equal volume of saturated ammonium sulfate solution and the sediment was dialysed with 0.01 M PBS (pH 7.4). All the reactions and dialysis should be protected away from exposure to light. The antibodies that conjugated with HRP were characterized by direct ELISA.

### 2.4. Sandwich ELISA

Ninety-six-well microplates were coated with anti-SEA mAb diluted in coating buffer (100 μL/well) and subsequently incubated at 4 °C overnight. After incubation, the wells were washed three times with washing buffer, and the free binding sites in the wells were blocked with blocking buffer (220 μL/well) for 2 h at 37 °C. After another washing step, 100 μL of a serially diluted SEA standard solution or sample extract solution was added to each well, and the microplate was incubated for 1 h at 37 °C. Afterward, 100 μL of HRP-labeled anti-SEA mAb was added to each well, and then the plates were incubated for 1 h at 37 °C. After the plate was washed five times, 100 μL of TMB substrate solution was added to each well, and was allowed to react with the labeled mAb at 37 °C for 15 min in the dark. The reaction was stopped by adding 2 M sulfuric acid (50 μL/well), and the absorbance was measured at 450 nm with a microplate reader. All measurements were performed in triplicate.

### 2.5. Indirect ELISA

Indirect ELISA was carried out to detect the serum titers and to screen the hybridoma cell lines. The ELISA plates with 100 μL/well of SEA in coating buffer were incubated at 37 °C for 2 h. After incubation, the plates were washed three times with washing buffer, blocked with blocking buffer (220 μL/well), and then incubated for 2 h at 37 °C. After washing, cell supernatant or mouse serum diluted with antibody dilution buffer was added to the wells (100 μL/well), and then the microplates were incubated at 37 °C for 30 min. After washing three times, HRP-labeled goat anti-mouse immunoglobulin previously diluted with antibody dilution buffer at a ratio of 1:3,000 was added (100 μL/well), and the plates were incubated at 37 °C for 30 min. After washing for four times, 100 μL of a freshly prepared TMB substrate solution was added to each well and allowed to react with the coating at 37 °C for 15 min in the dark. The reaction was stopped with 2 M sulfuric acid (50 μL/well), and the absorbance was measured at 450 nm with a microplate reader.

### 2.6. Pairwise Interaction Analysis

To select the best pair for the sandwich ELISA of SEA, mAbs from the 10 cell lines were labeled with HRP and analyzed in a sandwich ELISA format. One anti-SEA mAb was utilized as the capture antibody to pair with the other nine anti-SEA mAbs, which were labeled with HRP and used as the detection antibodies. Based on our experience, 4 μg/mL of capture antibody and 2 μg/mL of detection antibody yields optimal results; these amounts were therefore used in the subsequent experiments. SEA in PBS (100 ng/mL) was added to the positive control and 0.01 M PBS was added to negative control. The sandwich ELISA procedure was the same as described in subsection 2.4.

### 2.7. Preparation of Samples

Pure milk was purchased from a local market and was confirmed to be SEA-free by the China Entry-Exit Inspection and Quarantine Bureau (CIQ) in Jiangsu. The milk sample was centrifuged at 3,500 g at 15 °C for 10 min. After centrifugation, the milk fat was removed from the supernatant, and then the milk was diluted five-fold in water and analyzed immediately.

### 2.8. Recovery Analysis of Spiked Milk Samples by Sandwich ELISA

The milk was spiked with 0.5 ng/mL and 5 ng/mL of purified SEA. The spiked sample was treated as described in subsection 2.7. The SEA concentration in the spiked sample was calculated based on the standard curve and the SEA concentration in the undiluted sample was obtained by multiplying the concentration of the dilute sample by the dilution factor.

## 3. Results and Discussion

### 3.1. Production of Anti-SEA mAbs

Seven days after cell fusion, the mAb anti-SEA in the supernatant fluid from the cell culture plates was detected by indirect ELISA. Cells with high affinity for SEA were selected and subcloned three times by limiting dilution. After selection, 10 hybridoma cell lines designated as mAb1 to mAb10 and showing high affinity for SEA were obtained. These cells were intraperitoneally injected into mice, and ascitic fluid produced was taken for preparation of the mAbs.

### 3.2. Pairwise Interaction Analysis of the mAbs to SEA

**Figure 1:**
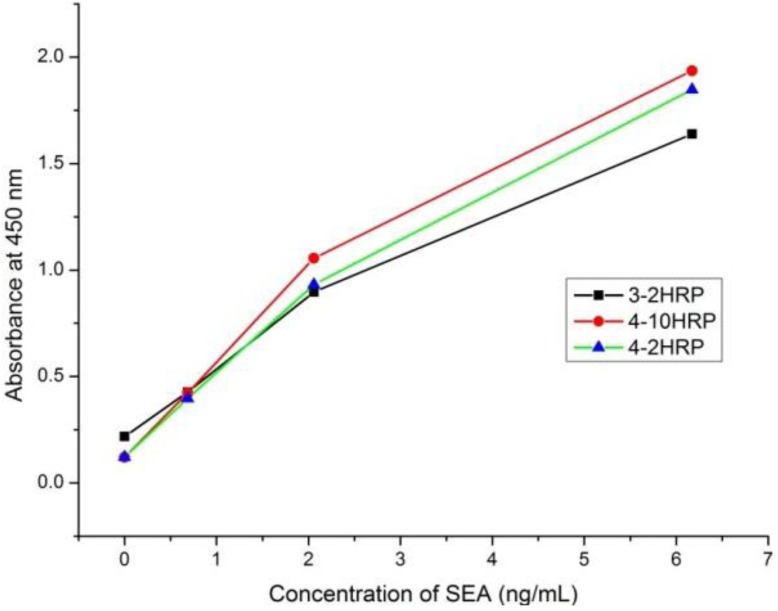
Comparsion of running curves in 0.01 M PBS between different pairs.

The ratios of OD_450_ for the positive control on the OD_450_ for the negative control are shown in Table 1. These results indicate that many pairs were obtained, and therefore, the mAbs recognizing various epitopes were well selected. However, the optimal combination is still unclear since the OD_450 _values of some positive control samples were >2.2; thus, 100 ng/mL SEA may be too high for the sandwich ELISA, and the pair with highest ratio may not guarantee the best sensitivity. Further experiments (Figure 1) confirmed that the lowest LOD was observed when mAb4 was chosen as the capture antibody and mAb10-HRP was used as the detection antibody.

**Table 1:** Sandwich ELISA for pair-wise interaction analysis (P/N value).

Detection mAb	Capture mAb									
	1	2	3	4	5	6	7	8	9	10
1-HRP	-	10.64	15.65	3.02	1.49	0.95	13.10	9.08	1.04	7.87
2-HRP	14.51	-	17.10	18.58	16.11	1.10	14.17	10.71	10.42	2.69
3-HRP	13.48	12.09	-	2.61	1.51	1.06	14.31	12.88	1.42	9.54
4-HRP	1.43	7.03	1.98	-	1.09	0.89	1.36	5.07	0.90	5.84
5-HRP	1.77	6.55	1.72	1.65	-	1.10	1.26	3.96	1.02	5.06
6-HRP	0.90	1.09	1.02	0.98	1.06	-	0.89	0.92	0.90	0.88
7-HRP	11.54	9.19	16.46	2.82	1.45	1.02	-	10.47	1.29	7.16
8-HRP	2.90	1.77	3.20	5.78	2.31	0.90	2.44	-	1.89	1.36
9-HRP	1.29	5.61	1.74	1.45	1.17	0.91	1.37	3.58	-	3.24
10-HRP	11.90	4.70	13.10	15.42	13.30	1.05	12.50	7.85	7.88	-

Note: P/N value was the ratio of optical density value of the sample to the negative control.

### 3.3. Development of mAb-Based Sandwich ELISA for the Detection of SEA

**Figure 2:**
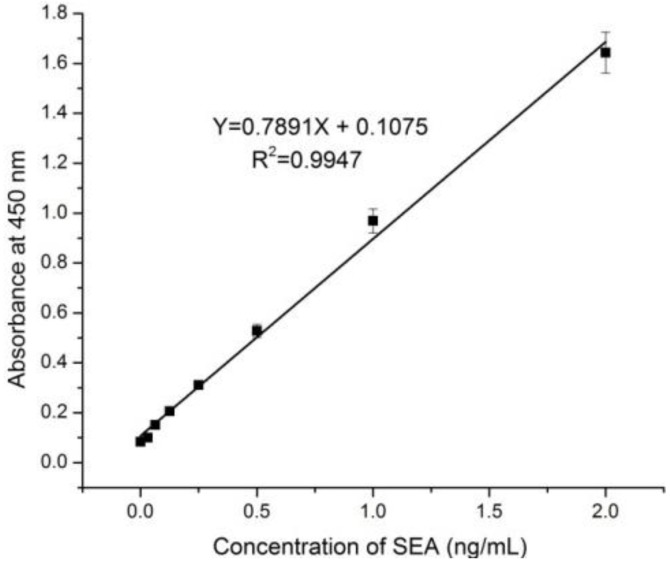
Standard curve of SEA in pure milk.

The sandwich ELISA for the detection of SEA was established using anti-SEA mAb4 (the capture antibody) and anti-SEA mAb10-HRP (the detection antibody). After optimization, the optimal concentration of the capture antibody and the dilution factor of the detection antibody were found to be 6 μg/mL and 1,400, respectively. Recombinant SEA was utilized as the standard, which was prepared as a solution in 0.01 M PBS containing 0.2% bovine serum albumin (BSA) and 0.5% Tween 20. The concentrations of BSA and Tween 20 were optimized for this dilution buffer to obtain a signal for the standard dilution buffer that is comparable to that for milk. Standard curve in pure milk is shown in Figure 2.

**Figure 3:**
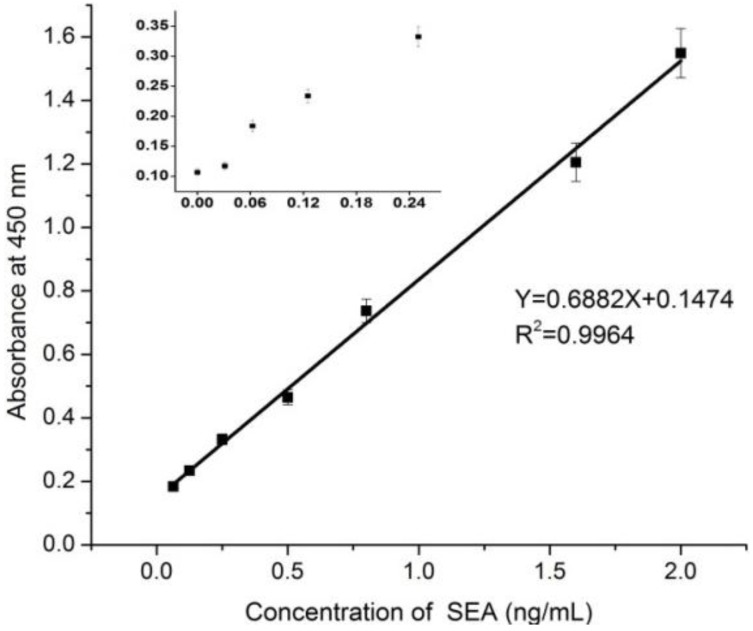
Calibration curve for the determination of SEA. Points were means of triplicate determinations. Small figure shows the signal of low concentrations from the calibration curve.

Figure 3 shows the calibration curve for the determination of SEA using the sandwich ELISA method developed in this study. The LOD was calculated as thrice the standard deviation of the blank value divided by the slope of the standard curve. The LOD of the method was determined to be 0.0282 ng/mL, and the linear dynamic range was found to be 0.06–2 ng/mL.

These values indicate that the method is sufficiently sensitive to detect SEA concentrations lower than 0.5 ng/mL, which is still a toxic level in milk based on some reports [27,28,29]. It also of interest that the LOD of the standard curve of SEA in 0.01 M PBS containing 0.2% BSA and 0.5% Tween 20 are lower than their counterparts in 0.01 M PBS. Furthermore, the signal of SEA in 0.01 M PBS decreased rapidly and was not very stable; the signal to noise ratio was 2.4 for 0.5 ng/mL SEA in 0.01 M PBS, whereas it was around 4.6 for the same amount in 0.01 M PBS containing 0.2% BSA and 0.5% Tween 20. In addition, the signal of the standard was enhanced when the BSA concentration was increased from 0% to 2%, but the background signal was also increased. However, the background signal decreased to acceptable levels when adding Tween 20. Signal enhancement by BSA was not clear, but the background signal probably decreased because Tween 20 could reduce nonspecific adsorption resulting from increasing concentrations of BSA.

### 3.4. Method Specificity

**Figure 4:**
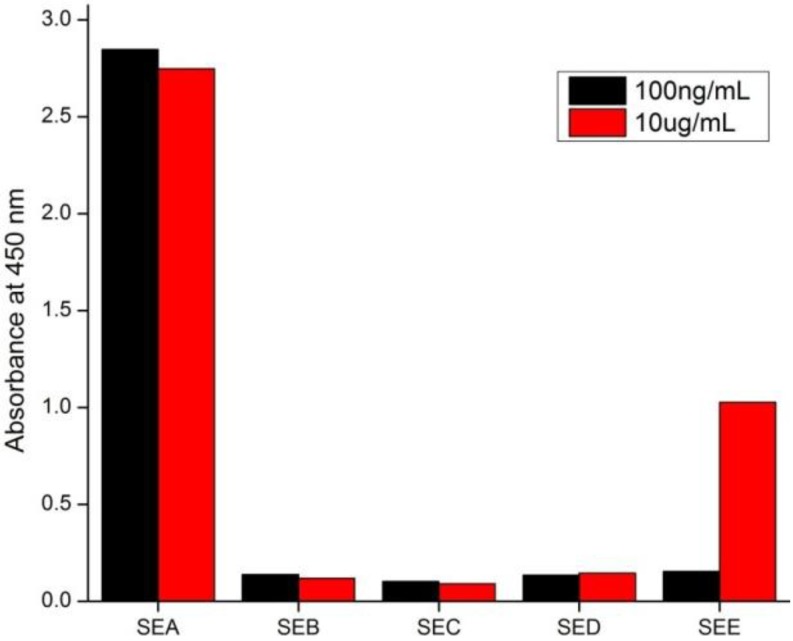
Cross-reactivities of the ELISA toward some types of SEs.

The specificity of ELISA to SEA should be evaluated because the various SEs share have a certain degree of homology. In the present study, solutions of SEB, SEC, SED, and SEE were prepared by dilution of measured amounts of the corresponding SEs in 10 μg/mL, 100 ng/mL, and 1 ng/mL of dilution buffer, and were subsequently analyzed by ELISA. A 0.01% cross-reactivity was observed with SEE when 10 μg/mL of each reactant was tested (Figure 4). However, no cross-reactivity was observed with SEB, SEC, SED, and SEE when the standard concentration was below 100 ng/mL. This finding is plausible since SEA and SEE share 70–90% sequence homology [14]. The aforementioned results therefore indicate that this method is highly specific for SEA. However, when commercial kits also utilizing mAbs to identify various SEs (such as the RIDASCREEN kit produced by R-Biopharm GmbH, Darmstadt, Germany) were used, the cross-reactivity between SEA and SEE was found to be approximately 10–20%. This difference in detected cross-reactivity may be related to the specificity of the antibodies used in the assays. 

### 3.5. Recovery Test and Validation Study

The intra- and inter-assay recovery and coefficient of variation (CV) of the ELISA method were evaluated next. The intra-assay recovery was determined by analyzing each concentration six times per run at one time, whereas the inter-assay recovery was determined by analyzing each concentration at six different times. Generally, recoveries of the analyte should be within 80% to 120% for an ELISA to be considered accurate, while the coefficient of variation (CV) of the analyte should be within 10% and 15% for the intra- and inter-assay precision, respectively. Results in Table 2 show that the intra- and inter-assay recoveries ranged from 82.67% to 108.93% and from 83.39% to 111.95%, respectively. The intra- and inter-assay CVs ranged from 3.16% to 6.05% and from 5.17% to 10.85%, respectively. These results indicate that this method gave accurate and repeatable results.

**Table 2:** Recovery of SEA from spiked milk by anti-SEA mAb 4 and mAb 10.

Spiked level (ng/mL)	Intra-assay (n = 6)		Inter-assay (n = 6)	
	Mean ±SD (ng/mL)	Recoveries (%)	Mean ±SD (ng/mL)	Recoveries (%)
5	5.446 ± 0.172	108.93	5.598 ± 0.289	111.95
0.5	0.413 ± 0.025	82.67	0.417 ± 0.045	83.39

## 4. Conclusions

In this research, a highly sensitive and specific mAb-based sandwich ELISA for the detection of SEA in food products was developed and validated. Ten hybridoma cell lines with high affinity were obtained after immunization, fusion, and selection. The best pair was optimized by pairwise analysis, and mAbs derived from the optimal pairs were used to develop the sandwich ELISA for SEA. The LOD of this ELISA method was determined to be 0.0282 ng/mL. To our knowledge, this is the most sensitive sandwich ELISA for SEA reported until now. Cross-reactivity of this sandwich ELISA for SEA with SEB, SEC, SED, and SEE was insignificant, and compared with some mAb-based commercial ELISA kits for SEA, this method is more specific. Recovery studies using pure milk showed that the method had high accuracy and reliability, therefore, the mAb-based sandwich ELISA developed in our study could be an effective method for routine monitoring of SEA in food samples.
